# Semantic processing during continuous speech production: an analysis from eye movements and EEG

**DOI:** 10.3389/fnhum.2023.1253211

**Published:** 2023-09-01

**Authors:** Jinfeng Huang, Gaoyan Zhang, Jianwu Dang, Yu Chen, Shoko Miyamoto

**Affiliations:** ^1^Faculty of Human Sciences, University of Tsukuba, Ibaraki, Japan; ^2^Research Institute, NeuralEcho Technology Co., Ltd., Beijing, China; ^3^Tianjin Key Laboratory of Cognitive Computing and Application, College of Intelligence and Computing, Tianjin University, Tianjin, China; ^4^Technical College for the Deaf, Tianjin University of Technology, Tianjin, China

**Keywords:** semantic processing, eye-voice span, continuous speech production, brain network, speech planning

## Abstract

**Introduction:**

Speech production involves neurological planning and articulatory execution. How speakers prepare for articulation is a significant aspect of speech production research. Previous studies have focused on isolated words or short phrases to explore speech planning mechanisms linked to articulatory behaviors, including investigating the eye-voice span (EVS) during text reading. However, these experimental paradigms lack real-world speech process replication. Additionally, our understanding of the neurological dimension of speech planning remains limited.

**Methods:**

This study examines speech planning mechanisms during continuous speech production by analyzing behavioral (eye movement and speech) and neurophysiological (EEG) data within a continuous speech production task. The study specifically investigates the influence of semantic consistency on speech planning and the occurrence of “look ahead” behavior.

**Results:**

The outcomes reveal the pivotal role of semantic coherence in facilitating fluent speech production. Speakers access lexical representations and phonological information before initiating speech, emphasizing the significance of semantic processing in speech planning. Behaviorally, the EVS decreases progressively during continuous reading of regular sentences, with a slight increase for non-regular sentences. Moreover, eye movement pattern analysis identifies two distinct speech production modes, highlighting the importance of semantic comprehension and prediction in higher-level lexical processing. Neurologically, the dual pathway model of speech production is supported, indicating a dorsal information flow and frontal lobe involvement. The brain network linked to semantic understanding exhibits a negative correlation with semantic coherence, with significant activation during semantic incoherence and suppression in regular sentences.

**Discussion:**

The study’s findings enhance comprehension of speech planning mechanisms and offer insights into the role of semantic coherence in continuous speech production. Furthermore, the research methodology establishes a valuable framework for future investigations in this domain.

## 1. Introduction

The speech planning process, based on the lexical access theory of speech production, consists of several stages of language processing, including lexical representation, semantic processing, and corresponding articulatory gestures. Typically, the process begins when speakers concentrate on a target concept and ends when they initiate the articulation (Levelt, 2001). Researchers have studied the mechanism of speech production both behaviorally and neurologically for many years with the aim of fully understanding the mechanism behind speech planning.

### 1.1. Investigation of speech production processing based on eye movement parameters

In behavioral studies, researchers have analyzed the latencies of speech planning in a speech production task by manipulating the syllable length, familiarity, and predictability of words (Meyer et al., 2003; Clifton et al., 2016; Robidoux and Besner, 2018; Shao et al., 2019; Lelonkiewicz et al., 2021). The results of these studies indicate that speech planning is mainly affected by syllable length and word familiarity, rather than semantics. To investigate the semantic implications, the researchers started using noun phrase reading tasks to examine the delay in speech planning. They combined eye movement with acoustic measures to speculate about potential speech planning processes through the behavioral analysis of eye movements (Levelt et al., 1999; Inhoff et al., 2011; Kirov and Wilson, 2013). For instance, in object naming tasks, speakers usually look at the first object for around 500–700 ms, then begin inspecting the second object, and about 150–350 ms later, they initiate the articulation of the first object (Wilshire and Saffran, 2005; Meyer et al., 2007; Cholin et al., 2011). This indicates that when the eyes move from the current word to the upcoming word, there is a temporal overlap between the fixation of the upcoming word and the speech onset of the current word. These phenomena are interpreted as speakers looking ahead several words for the integrated processing of the current words. This phenomenon only occurs when the speaker can anticipate upcoming sounds by “looking ahead” in context (Dell, 1986). In recent years, numerous studies have started to investigate changes in the “eye-voice span (EVS)” of speech planning in continuous speech by asking participants to read sentences (Inhoff et al., 2011; Laubrock and Kliegl, 2015; Huang et al., 2018). In the second half of a sentence, as the speaker reads, the onset of uttering a word gradually approaches the onset of moving the eyes from the word. This results in speech planning not requiring “looking ahead” in the latter section of the sentence. This is probably due to the coherent contextual semantics of continuous speech production. In a sentence, the preceding words can provide helpful semantic information that can aid in understanding or predicting the meaning of upcoming words.

### 1.2. Neural model of language cognitive processing

Although behavioral studies may not offer ample explanations for cognitive processes during latent time, speech processing involves several complex neural interactions. Consequently, several researchers explored neurological aspects of speech processing. The Wernicke-Geschwind Model (Damasio and Geschwind, 1984; Tremblay and Dick, 2016) is a classic neural model that explains a single pathway of speech production, which involves (i) form-sound matching of visual word recognition in the left superior angular gyrus; (ii) auditory speech recognition in the left middle temporal cortex; (iii) semantic comprehension in the posterior cortex of the left temporal lobe (posterior Wernicke Area); (iv) motor planning of speech production by the Broca Area in the posterior inferior cortex of the left frontal lobe; and (v) articulatory motor control by the primary motor cortex in the central anterior gyrus. This model anatomically confirmed the importance of both semantic comprehension and phonological encoding as prerequisites for speech production. In contrast to the Wernicke-Geschwind model, a neuroanatomical model of speech production proposed by Hickok and Poeppel emphasizes dual route processing (Hickok and Poeppel, 2004, 2007; Sammler et al., 2015). The model consists of a ventral stream and a dorsal stream. The ventral stream extends bilaterally from the posterior middle and inferior temporal gyrus to the anterior middle temporal gyrus, supporting auditory comprehension. The model also suggests the presence of a left-lateralized sensorimotor dorsal stream involving the superior temporal gyrus at the notch of the Sylvian fissure, as well as the posterior inferior frontal gyrus and lateral premotor cortex. According to this model, the dorsal stream is responsible for the articulatory behavior involved in speech production, while the ventral stream is responsible for semantic comprehension. More recently, Arbib (2017) attempted to integrate functional networks involved in language processing in the brain using a dual-stream model of visual and auditory processing. They found that when language comprehension operates at the level of using words as symbols, most of the syntactic and semantic processing is provided by the ventral pathway. On the other hand, the dorsal pathway functions when words are perceived and produced as objects of articulatory behavior (Arbib, 2017). These findings further support the dual-stream model of speech production and demonstrate that the two streams play distinct roles in the processing of semantic comprehension and articulatory movements. However, during the process of reading, as the number of words increases, the neural activities of different functional modules, including language information representation, semantic understanding, and phonetic planning, overlap and form a complex brain network (Schoffelen et al., 2017). As a result, the two-stream model encounters challenges in accurately describing the intricate and dynamic nature of this process, particularly in terms of the need for rapid comprehension and integration of continuous contextual semantics. It is possible to speculate that there might be varying degrees of emphasis in the activation of different pathways in the brain based on the level of difficulty in semantic comprehension. To gain insights into the underlying brain activity, it is necessary to investigate this by controlling for the semantic coherence of sentences.

In addition, most models of speech production are based on anatomy and functional magnetic resonance imaging (fMRI) functional networks. However, fMRI’s inferior time resolution prevents it from accurately demonstrating the interaction and dynamic changes between various brain regions that occur during speech processing, which often involves complex interactions between neurons that happen on a millisecond timescale (Carreiras et al., 2014). Studies show that speech planning happens with interactions among functionally and/or structurally linked brain regions that are spatially separated (Rubinov and Sporns, 2010; Park and Friston, 2013). Noninvasive electroencephalography (EEG) can fully record the dynamic interactions between neurons with sub-millisecond resolution and can trace the neural processes involved in speech production, from low-level visual perception to word meaning, through appropriate designs. Furthermore, high-density EEG analysis technology can infer the source of the signal in the brain from scalp multi-channel electrical signals (Chang et al., 2018). Combining EEG with prior knowledge obtained from fMRI can comprehensively investigate the temporospatial brain process involved in continuous speech production (Zhao et al., 2017). Therefore, this study used a multimodal data analysis method that combined EEG, eye-tracking, and speech data during oral reading of continuous sentences to examine the speech planning process from a human behavioral and neurological perspective. We expect that these methods will help us uncover the causes of the speech planning process mechanism from semantic processing.

In this study, our aim is to investigate the mechanisms underlying semantic processing in speech planning by using a multimodal analysis that integrates human behavior and neurology. Our methodology involves the simultaneous use of electroencephalography (EEG), eye-tracking, and speech behavioral data collected during the oral reading of continuous sentences. The central objective of our research is to examine how the semantic coherence within sentences affects speech planning in continuous speech production. To achieve this, we have designed an experiment that includes both regular sentences, which contain coherent contextual semantic information, and non-regular sentences that lack such information. This manipulation involves altering the word order within sentences. Given the crucial role of contextual semantic information in facilitating smooth and coordinated speech production, we hypothesize a significant correlation between anticipatory eye monitoring and semantic anticipation. This discovery could enhance the comprehension of the dual-stream model by establishing a clearer link between eye movement patterns and neural activity during speech production. Our analysis incorporates two primary approaches: a behavioral analysis using eye-movement metrics and a time-frequency dynamic brain network analysis based on EEG data. We propose the hypothesis that as participants comprehend semantic information coherently, the extent of anticipatory eye movements, indicating look-ahead behavior, will gradually decrease. This reduction in look-ahead magnitude is expected to stabilize within a certain range throughout the reading process. Additionally, we suggest that heightened semantic association suppresses the activation of semantic processing networks. Conversely, the presence of semantic inconsistency amplifies the degree of look-ahead, although its impact on anticipatory effects diminishes. These intriguing observations will provide insights into the activation of the brain’s semantic processing network, thereby complementing the dual-stream model of how semantic and non-semantic pathways coordinate speech production. Therefore, this study contributes to a deeper understanding of the intricate interplay between semantic processing, anticipatory behaviors, and the neural processes involved in speech production.

## 2. Materials and methods

### 2.1. Participants

A total of 38 graduate students, 26 male and 12 female, with a mean age of 25.0 years (SD = 1.8, range 22–29), were recruited for this study from JAIST and Tianjin University. All participants were native Chinese speakers with no history of reading difficulties, and had normal or corrected-to-normal visual acuity (Strasburger et al., 2011) and normal hearing and speaking abilities. They were assessed as right-handed using a modified Edinburgh handedness test (Oldfield, 1971), and reported no neurological or psychological disorders. Pronunciation and eye movement tests were conducted, ensuring all participants had the same level of understanding of the experiments. Participants provided written informed consent, and were paid for their participation and informed of their right to terminate their participation at any time. All participants then took part in the sentence oral reading task after passing all tests and signing relevant documents. Technical problems, disorders, or incorrect stimulus reading led to exclusion of data from six participants. Ethical approval was obtained from the JAIST and Tianjin University Research Ethics Committee.

### 2.2. Language materials

For this experiment, we specifically designed two types of sentences: regular sentences (RS) and non-regular sentences (NRS), each containing 60 sentences. RS contain normal semantic coherence, meaning their content is coherent and easily understood. Conversely, NRS sentences were created by randomly rearranging the order of words in RS and adjusting them. While each word in NRS sentences is individually understandable, the sentence as a whole does not make sense. To control for word-length effects on speech production, all sentences contained 8 disyllabic words with clear grammatical boundaries. Additionally, all disyllabic words were high-frequency words, ranging from 163 to 4,243 occurrences per million (Cai and Brysbaert, 2010). Table 1 provides examples for each sentence type.

**Table 1:** Example stimuli.

Condition	Example
RS	天气 预报 报道 天津 天气 凉爽 适合 旅游。 The weather forecast reports cool weather in Tianjin, suitable for travel
NRS	旅游 天津 预报 适合 天气 报道 凉爽 天气。 Travel in Tianjin forecast suitable for the weather reports cool weather

The example shows disyllabic word boundaries by spaces, which are not present in the actual experiment.

### 2.3. Multimodal data acquisition

The study was conducted within an electromagnetically shielded room, utilizing a Philips 246V5LHAB monitor with a 1,920 × 1,080 resolution and 60 Hz refresh rate. Eye movements of the participants were binocularly recorded using an infrared video-based eye tracking system (Eyelink 1000 Plus, SR Research Ltd., Ontario, Canada) at a sampling rate of 1,000 Hz. Concurrently, speech was recorded via a Rode NTG-3 microphone, with a sampling rate of 44,100 Hz. EEG data was collected using an elastic cap equipped with 128 Ag/AgCl scalp electrodes, referencing left and right mastoids online and offline, and with a ground electrode located at AFz. The horizontal electrooculogram (EOG) was measured with 2 electrodes at the outer canthi. The vertical EOG was measured with 4 electrodes placed above and below both eyes. All impedances are adjusted to below 5kΩ. The recorded data was amplified using a SynAmps RT amplifier (Neuroscan, United States) with a 1,000-Hz sampling rate. Participants were asked to place their foreheads in a fixator to secure their heads throughout the experiment recording.

**Figure 1:**
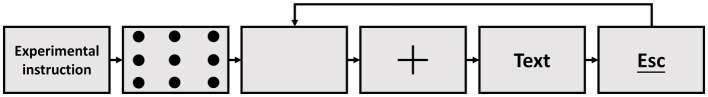
The procedure used in the present study.

A system for measuring eye movements, speech, and EEG was designed to conduct the experiment using MATLAB (version: 2017a) and PsychToolbox (version: 3.0.14) and simulate natural reading conditions. The experiment began with a nine-point calibration routine before each trial to ensure gaze accuracy deviation was less than 0.50. Figure 1 shows that each trial started with a 2,000-ms resting period, followed by a fixation cross that appeared at the screen center for 1,000 ms. Then, a randomly selected sentence was displayed at the center of the monitor. The eye movement and speech data were recorded separately for each sentence, starting synchronously when the cross disappeared and stopping when the participant pressed the ESC key to finish reading. The EEG data were recorded from the beginning of the experimental instruction and continued until all trials were completed. Time synchronization between EEG and behavioral data was achieved using Triggers. Each time the cross symbol disappeared, the system sent a Trigger with the number 255 to the EEG waveform data, indicating the start of the current trial. Similarly, when the ESC button was triggered, the system sent Triggers of number 128 to the EEG data, indicating the end of the current trial. During this entire period, if the participant’s fixation point first appeared in any of the 8 words fields, a corresponding numbered trigger was marked on the EEG signal. Each participant completed two experiments, the RS experiment, and the NRS experiment. Each experiment consisted of three blocks of 60 randomly chosen sentences. The duration of the procedure per participant ranged from 52 to 88 min, with simultaneous recording of EEG, eye movement, and speech data for the entire experiment. To ensure the stability of the oculomotor parameters during the experiment, the oculomotor parameters will be corrected again every tenth trails. We have also set up four buttons. They are Exit Experiment Program (Esc), Proceed to Next Trial (Enter), Tentative Trial (Space) and Return to Previous Trial (Backspace). When the experiment is tentatively resumed, the eye movement parameters will be recalibrated to eliminate the eye movement drift that occurs after rest.

### 2.4. Eye movement and speech data analysis

In this study, we monitored both eyes during reading and used the average of the left and right eye movements as the data. Prior to statistical analysis, we screened the eye-tracking record of each participant to remove erroneous fixations, blinks, and fixations that were very brief or long (less than 50 ms or greater than 750 ms) (Clifton et al., 2016). The horizontal position of the gaze was mapped to words positions, and standard measures were determined such as First Fixation Duration (FFD; duration of the first fixation on a words in first-pass reading), Mean Fixation Duration (MFD; mean of all duration of fixations on a words in first-pass reading), Gaze Duration (GD; sum of all duration of fixations on a words in first-pass reading), as well as skipping probability, single-fixation probability, refixation probability, and regression probability. All these metrics were used to measure behavioral outcomes. We also extracted the gaze onset time (GOn) and offset time (GOf) of every word in the sentence according to the eye movement trajectory. Only the first-pass reading, from the entry of the gaze point to the exit, was considered while regression and second-pass readings were disregarded. Additionally, SPPAS (Speech Phonetization Alignment and Syllabification), a speech automatic annotation and analysis toolbox, was employed to detect speech endpoints and to extract the pronunciation onset time and pronunciation offset time of every word. Moreover, the speech pronunciation onset time (SOn) and offset time (SOf) of each word in the continuous speech were also obtained. Finally, the eye-voice span (EVS) is calculated by the time difference between GOn and SOn (Laubrock and Kliegl, 2015).

In addition, we divided speech production into three different periods to be explored based on four time points: GOn, GOf, SOn and SOf. These periods consist of (i) the viewing period (VP) between GOn and GOf, (ii) the overlapping period (OP) between GOf and SOn, and (iii) the speech period (SP) from SOn to SOf. Together, VP and OP constitute the EVS.

### 2.5. EEG data analysis

#### 2.5.1. EEG data pre-processing

The EEG recordings were pre-processed using the EEGLAB toolbox (Delorme and Makeig, 2004). Initially, a 1 Hz high-pass filter was employed, in order to eliminate DC offset or slow drift potential (<1 Hz). To reduce noise and interference from extraneous data, the original sampling rate was reduced from 1,000 Hz to 250 Hz. A low-pass filter with a cut-off frequency of 60 Hz was then used to retain the high gamma component involved in speech feedback. Additionally, a band-notch filter ranging from 49 to 51 Hz was applied to remove industrial frequency interference at 50 Hz. Defective channels with more than 10% anomalous fluctuations were removed prior to the averaging of the data. The continuous data was then divided into 180 epochs, each ranging from −1,000 ms to the end of utterance of each sentence at around 5,019 ms in the RS and 5,108 ms in the NRS, with the 1,000 ms pre-onset period serving as a baseline. The *clean_rawdata()* plugin in EEGLAB was utilized to de-noise continuous EEG channel data (Mullen et al., 2015).

Independent Component Analysis (ICA) was used to separate brain and non-brain effective source processes from the continuous EEG data (Delorme and Makeig, 2004; Chaumon et al., 2015). ICA effectively identified signal sources independent of each other in multichannel EEG data and projected them onto scalp electrodes. We applied the adaptive mixture independent component analysis algorithm (AMICA) to transform the scalp-EEG data from a channel basis to a component basis (Hsu et al., 2018) and separated those maximally independent cortical sources from biological artifacts and noise components (Chaumon et al., 2015; Pion-Tonachini et al., 2019). After rejecting noisy AMICA components, an equivalent current dipole (ECD) model of each brain independent component (IC) was computed using the standard boundary element method (BEM) head model embedded in the EEGLAB DIPFIT plug-in to localize dipolar sources on the cortex. The ICLabel plug-in applies a seven-class categorization for the independent components (IC) based on their spatiotemporal features, consisting of Brain, Muscle, Eye, Heart, Line Noise, Channel Noise, and Other, estimated by an expert (Pion-Tonachini et al., 2019). In this study, we focused on brain-effective components, identifying the components with Brain label probability >0.7 and residual <0.15, while disregarding the rest automatically.

#### 2.5.2. Group-level effective connectivity analysis

The source information flow toolbox (SIFT) was used in this paper to model continuous or event-related effective connectivity between ICs time series (Delorme et al., 2011; Mullen, 2014). During preprocessing, the data was epoched to −1,000 to 5,019 ms for the RS and −1,000 to 5,108 ms for the NRS relative to stimulus onset. A sliding window length of 250 ms, a window step size of 25 ms, and 60 bins of logarithmic frequency ranging from 1 to 60 Hz were used. The Vieira-Morf algorithm was employed to fit a linear vector adaptive multivariate auto-regressive (AMVAR) model (Ding et al., 2000). Following model fitting, stability, and residual whiteness tests, a multivariate method based on Granger causality (dDTF08, direct Directed Transfer Function) (Korzeniewska et al., 2008) was estimated from the AMVAR coefficients to determine multivariate information flow (Ding et al., 2000; Schelter et al., 2009; Blinowska, 2011). Connectivity measures were then propagated to the group level and a statistical analysis was performed using a group-SIFT method (Loo et al., 2019). To achieve spatial coherence of EEG dynamics, a Network Projection approach was utilized, similar to the voxel-by-voxel analysis in functional magnetic resonance images (fMRI). A 3-D Gaussian kernel was used to model each dipole, transforming their locations into probability density of dipoles. This was defined by full width at half maximum (FWHM) on a substrate of 76 anatomical regions of interest in the brain based on the Automated Anatomical Labeling (AAL) atlas. The names of the 76 anatomical regions of interest and their coordinate location parameters are listed. Next, an anatomical ROI-to-ROI pairwise weighted dipole density, weighted with dDTF08, was calculated, generating a connectivity matrix with 76 × 76 edges of the graph. Each participant was associated with a four-dimensional tensor of ROI origins, ROI destinations, frequency bins, and time points. The tensors of individual participants were concatenated for each group, on which edges of the graph were selected. Intergroup comparison was performed on overlapping edges of the graphs between the groups using a two-sample *t*-test for both ERSP (event-related spectral perturbation) and weighted dDTF08 to generate the initial masked t-score maps with an uncorrected *p* < 0.05. Finally, group differences in the temporal dynamics and spatial structure of event-related effective connectivity were explored.

#### 2.5.3. Dynamic time-frequency correlation analysis

We investigate the impact of reading sentences on the network of brain functions involved in speech planning to assess changes in brain activity. To enhance the spatial resolution of our EEG analysis, we utilized the pre-existing fMRI realizations of brain networks as both the initial values and constraints. The fMRI database of the Atlas of Human Brain Functions (available at Brainnetome atlas: http://atlas.brainnetome.org/index.html) was utilized in this study to generate functional adjacency matrices based on the semantic brain functions (Fan et al., 2016). Firstly, filter out all brain regions in the Brainnetome database that are relevant to language functionality. Then, retain all brain regions within the language-related areas that have close connections to semantics. Construct a semantic functional brain network using these identified brain regions. Finally, we calculated similarities between each function using both fMRI-based functional adjacency matrices (FAM) and EEG-based FAMs simultaneously, employing the Pearson correlation coefficient. Using this approach, we computed the correlation between the fMRI-based FAM and the time-varying FAM obtained from our EEG experiment, resulting in a time-varying correlation coefficient that characterizes the activity of the cerebral network. As the coefficient increases during a specific time interval, the corresponding brain network exhibits greater activation.

## 3. Results

### 3.1. Behavioral results

**Figure 2:**
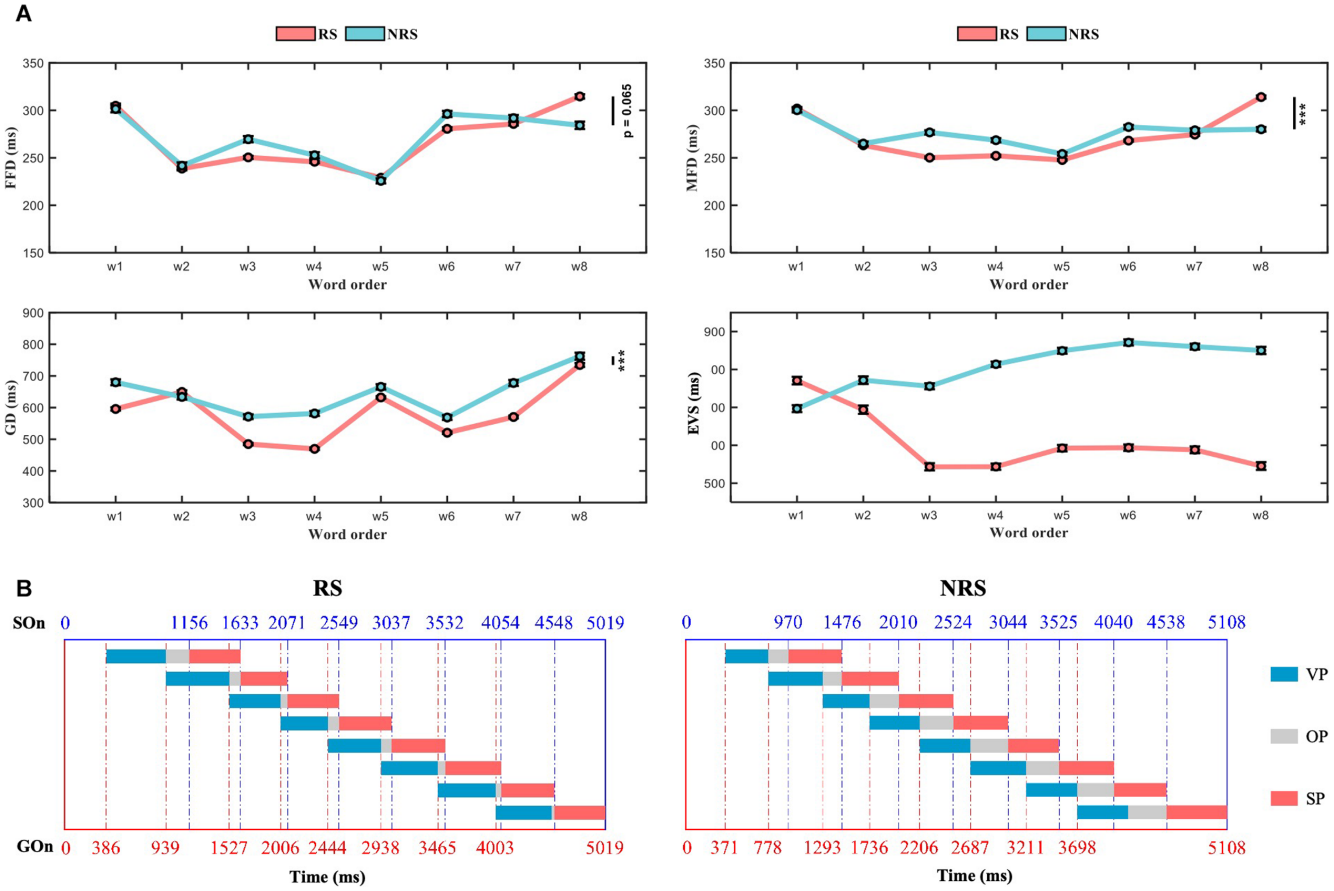
Schematic diagram of the variation in the duration of VP, OP, and SP for the RS and NRS groups. **(A)** The graph illustrates the average trends of FFD, MFD, GD, and EVS for both the RS and NRS groups. **(B)** Depicts the variation in schematic diagrams for the duration of VP, OP, and SP in both the RS and NRS groups.

Excluding 12 subjects whose eye movement data and voice data were lost during the recording process, a total of 26 subjects ‘data were analyzed. Table 2 summarizes the general descriptive statistics related to eye movement and pronunciation during oral reading. The reading time for the RS group was 5,019 ms (sd = 671), and 5,108 ms (sd = 1,007) for the NRS group. After multiple comparisons, no significant difference was found between the two groups (*F* = 3.01, *p* = 0.083). Moreover, the required time to produce words was almost identical in both groups, with 314 ms (sd = 13) for RS and 319 ms (sd = 7) for NRS. Overall, there was no significant difference in FFD between RS and NRS (*F* = 3.4, *p* = 0.065). However, there were significant differences in MFD (*F* = 26.31, *p* < 0.001), GD (*F* = 344.17, *p* < 0.001), and EVS (*F* = 583.05, *p* < 0.001). The result of the RS, the EVS of the initial word is 770 ms (sd = 280) and decreases gradually to 545 ms (sd = 239) for the last word. As one can see, the time difference of the EVS between the initial word and the last word reached −225 ms although all of the word lengths were the same. On average, the EVS of the last word is about 29% shorter than that of the initial one. It indicates that the EVS of speech planning changes with the location of the words in continuous speech, and this change continues to the third word. Starting from the third word, the change in EVS has become smaller and gradually stabilized. In addition, the EVS of the third word is 544 ms (sd = 224) and has no significant difference with the last word (*F* = 0.1, *p* = 0.751). In contrast, for the NRS, the EVS of initial word is 599 ms (sd = 241) and the last word is 840 ms (sd = 282). The average EVS of the initial word and the last word differs by 241 ms, has significant difference (*F* = 34.99, *p* = 0.000). Moreover, the number of words in the sentence increased, the EVS increased slightly and there was no significant difference from the third word onwards. The average trends for FFD, MFD, GD, and EVS are illustrated in Figure 2A.

**Table 2:** Descriptive statistics for RS and NRS oral reading.

		W1	W2	W3	W4	W5	W6	W7	W8
FFD [ms]	*RS*	305 (152)	239 (135)	250 (111)	246 (98)	229 (87)	281 (110)	286 (117)	315 (155)
	*NRS*	301 (146)	242 (147)	270 (137)	253 (134)	226 (110)	296 (133)	292 (138)	284 (157)
MFD [ms]	*RS*	302 (149)	263 (134)	250 (110)	252 (109)	248 (105)	268 (115)	275 (124)	314 (163)
	*NRS*	300 (146)	265 (143)	277 (137)	269 (136)	254 (127)	282 (133)	279 (139)	280 (156)
GD [ms]	*RS*	596 (331)	649 (348)	485 (253)	470 (265)	632 (263)	521 (264)	570 (295)	735 (407)
	*NRS*	680 (382)	634 (347)	571 (308)	581 (326)	666 (345)	569 (308)	678 (385)	762 (449)
EVS [ms]	*RS*	771 (280)	694 (289)	543 (224)	544 (194)	592 (207)	594 (209)	588 (217)	545 (239)
	*NRS*	697 (241)	772 (280)	756 (217)	814 (202)	849 (226)	871 (240)	860 (227)	850 (282)
Number of fixations	*RS*	1.72 (1.20)	2.43 (1.21)	1.91 (0.94)	1.83 (0.97)	2.53 (1.01)	1.90 (1.01)	2.02 (1.05)	2.13 (1.37)
	*NRS*	2.09 (1.29)	2.30 (1.19)	1.97 (1.06)	2.08 (1.11)	2.54 (1.25)	1.91 (1.07)	2.29 (1.32)	2.47 (1.59)
Skipping probability	*RS*	0.13	0.01	0.01	0.02	0.01	0.02	0.03	0.09
	*NRS*	0.08	0.04	0.05	0.04	0.03	0.05	0.06	0.09
Single-fixation probability	*RS*	0.41	0.18	0.34	0.39	0.12	0.35	0.30	0.28
	*NRS*	0.30	0.19	0.31	0.28	0.15	0.33	0.24	0.20
Refixation probability	*RS*	0.06	0.45	0.19	0.23	0.73	0.07	0.08	0.05
	*NRS*	0.07	0.38	0.22	0.31	0.62	0.10	0.11	0.04
Regression probability	*RS*	0.09	0.49	0.22	0.24	0.73	0.08	0.11	0.07
	*NRS*	0.09	0.43	0.26	0.36	0.63	0.15	0.17	0.05
Time to word pronunciation [ms]	*RS*	553 (370)	589 (298)	478 (220)	439 (212)	493 (220)	528 (234)	537 (258)	517 (87)
	*NRS*	408 (285)	515 (301)	443 (237)	470 (213)	480 (259)	524 (289)	488 (285)	475 (107)

On average, the number of fixations in NRS was significantly higher than in RS (*F* = 170.81, *p* < 0.001). The RS group had a higher probability of skipping the first word compared to the NRS group, while the skipping rate of subsequent words was higher in the NRS group with the same final word. The single-fixation probability of RS was higher than NRS. The probability of refixations and regressions showed the same trend.

When examining three distinct periods of speech production, the statistical analysis revealed significant differences between the RS and NRS groups in terms of the mean values of VP. For the RS group, the mean VP was 517 ms (sd = 87), while for the NRS group, it was 475 ms (sd = 107) (*F* = 41.15, *p* < 0.001). Similarly, the mean OP showed a significant difference between the two groups, with 92 ms (sd = 143) for the RS group and 293 ms (sd = 265) for the NRS group (*F* = 315.38, *p* < 0.001). Furthermore, the mean SP differed significantly between the RS group (483 ms, sd = 72) and the NRS group (517 ms, sd = 140) (*F* = 35.45, *p* < 0.001). The mean EVS statistics were also significantly different, with 608 ms (sd = 114) for the RS group and 809 ms (sd = 104) for the NRS group (*F* = 583.05, *p* < 0.001).

Subsequent separate statistical analyses were conducted for GD, OP, SP, and EVS for each word in the sentences of both groups. In the RS group, the GD and SP of each word exhibited stable distributions ranging from 430 ms to 590 ms (average = 517 ms, sd = 87 ms; average = 483 ms, sd = 72 ms). The latency of EVS for pronunciation demonstrated two distinct descending phases: the first stage encompassed the first word (average = 770 ms, sd = 280 ms) to the fourth word (average = 543 ms, sd = 194 ms), while the second stage spanned from the fifth word (average = 593 ms, sd = 207 ms) to the last word (average = 545 ms, sd = 239 ms). Upon closer examination, it becomes apparent that the difference in EVS can be largely attributed to the decrease in OP, indicating an overall decreasing trend. In the NRS group, both GD and SP exhibited stable distributions between 400 ms and 570 ms for each word (average = 475 ms, sd = 107 ms; average = 517 ms, sd = 140 ms). Similarly, the EVS latency for pronunciation displayed two stages: an ascending stage from the first word (average = 599 ms, sd = 241 ms) to the fifth word (average = 838 ms, sd = 226 ms), followed by a smooth phase from the sixth word (average = 838 ms, sd = 240 ms) to the last word (average = 840 ms, sd = 282 ms). Once again, this difference in EVS can be largely attributed to the decrease in OP, with an overall upward trend.

This figure illustrates the distribution of the mean VP, OP, and SP for a sample of 180 sentences across 26 topics as shown in Figure 2B. The left and right figures, respectively, represent the RS and NRS conditions. The horizontal axis of the figure indicates the time scale, while the rectangle whose color varies across time reflects a clear visualization of duration and variation for the two groups under different conditions. The boundary between GOn and GOf is represented by the red dashed line, while the blue dashed line represents the boundary between SOn and SOf. The blue, gray, and red squares represent the duration of VP, OP, and SP, respectively. The duration of EVS equals the total duration of VP and OP.

### 3.2. EEG results

**Figure 3:**
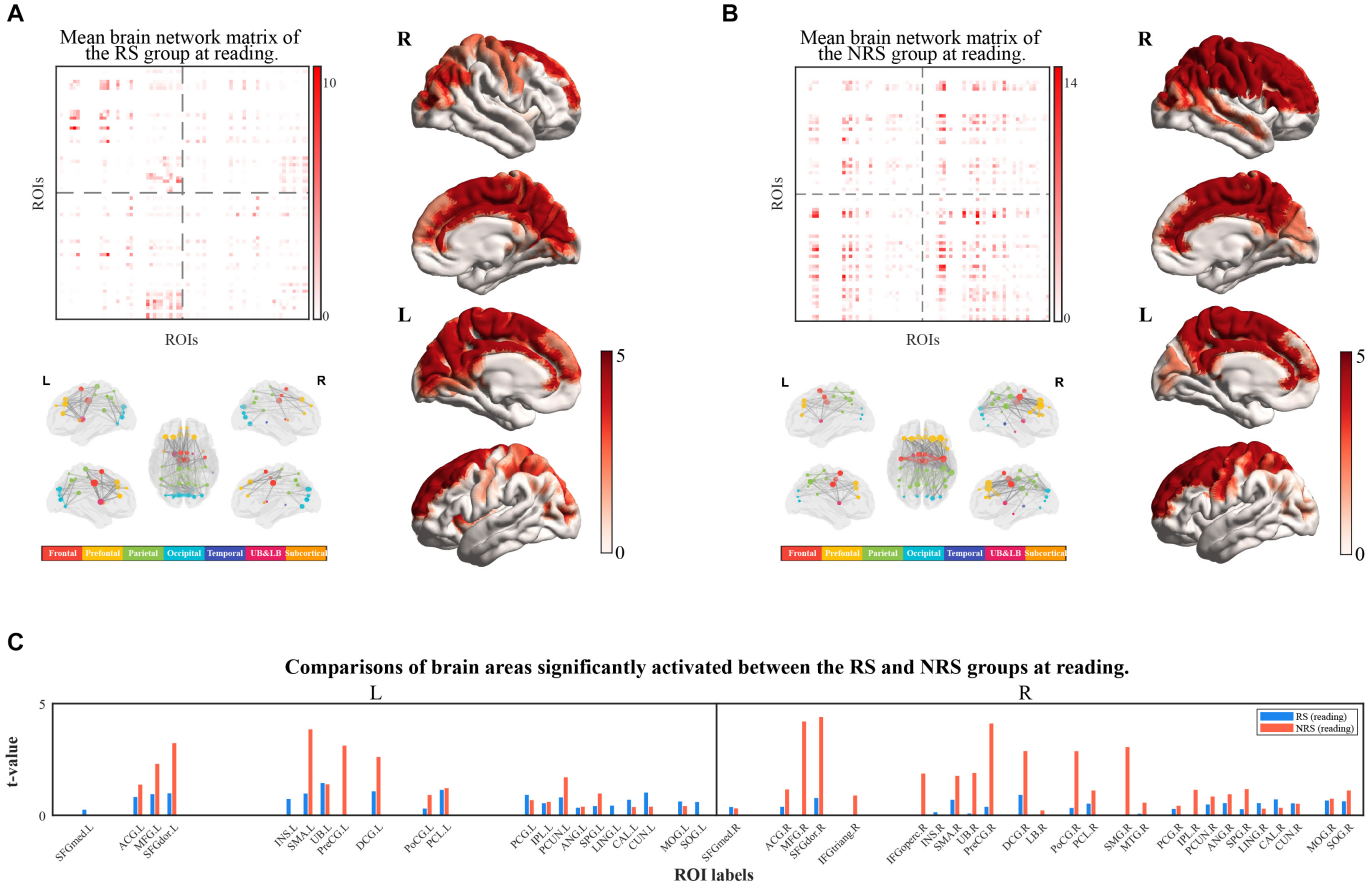
**(A)** Shows the results of the brain networks matrix (top-left view), brain networks (down-left view), and cortical localization (right view) under RS tasks at the reading period; **(B)** shows that the results of the brain networks matrix (top-left view) brain networks (down-left view) and cortical localization (right view) under RS tasks at reading period; **(C)** shows the comparison of activated brain regions in the RS and NRS at reading period, with the RS group in blue and the NRS group in red, and differentiates between the left and right brains.

The brain networks constructed from source space were mapped to the corresponding brain cortex, and the activated brain areas underwent statistical analysis. For significant edge values and cortical distribution, masking was applied on the distribution of the *t*-values associated with event-related effective connections, with *p* < 0.05. The effect of the baseline was eliminated in both groups of sentences during the reading period. Figure 3 illustrates the results of reading aloud for both the RS and NRS groups. The edge matrix of the brain network, the cerebral network node, visualization of the edge in 5 views, and topography of the activated brain areas in 4 views were visualized separately for each data set. The right bars in matrices and topographic maps correspond to the *t*-values obtained after a two-sample *t*-test based on significance. Nodes in the brain network were categorized into seven different colored brain regions, as shown in the accompanying color bar diagram.

The results of the RS group during reading are displayed in Figure 3A. The study findings indicate the activation of 40 brain regions during oral reading. The connectivity between brain regions remains high through the visualization of brain network edge matrix and cortical visualization. A distinct dorsal pathway from the occipital lobe to the frontal lobe is evident. The visualization of the brain network reveals significantly more active connections between brain regions in the left hemisphere compared to the right hemisphere, indicating a pronounced left hemisphere bias. Figure 3B depicts the activation of 42 brain regions during reading in the NRS group. In comparison to the RS group, the brain network exhibits significantly higher connectivity strength and stronger cortical activation. Specifically, the right hemisphere regions, such as the superior parietal lobule, the central anterior and posterior gyri, and the frontal lobe, demonstrate stronger activation compared to the RS task, specifically in a substantial portion of the right frontal lobe. Brain network visualization depicts a markedly enhanced connectivity between the left and right hemispheres in the NRS group, demonstrating a bias toward the right hemisphere. Figure 3C presents a comparison of the brain regions’ activation during reading in the two groups of sentences. The results indicate a more balanced activation distribution between the left and right hemispheres in the RS group, with stronger connectivity among brain regions in the left hemisphere. In contrast, the NRS group demonstrates a distinct advantage in the right hemisphere, particularly with stronger activation in the right frontal and parietal regions. Brain network analysis reveals that the NRS group exhibits greater complexity and stronger connectivity in multiple brain regions than the RS group.

**Figure 4:**
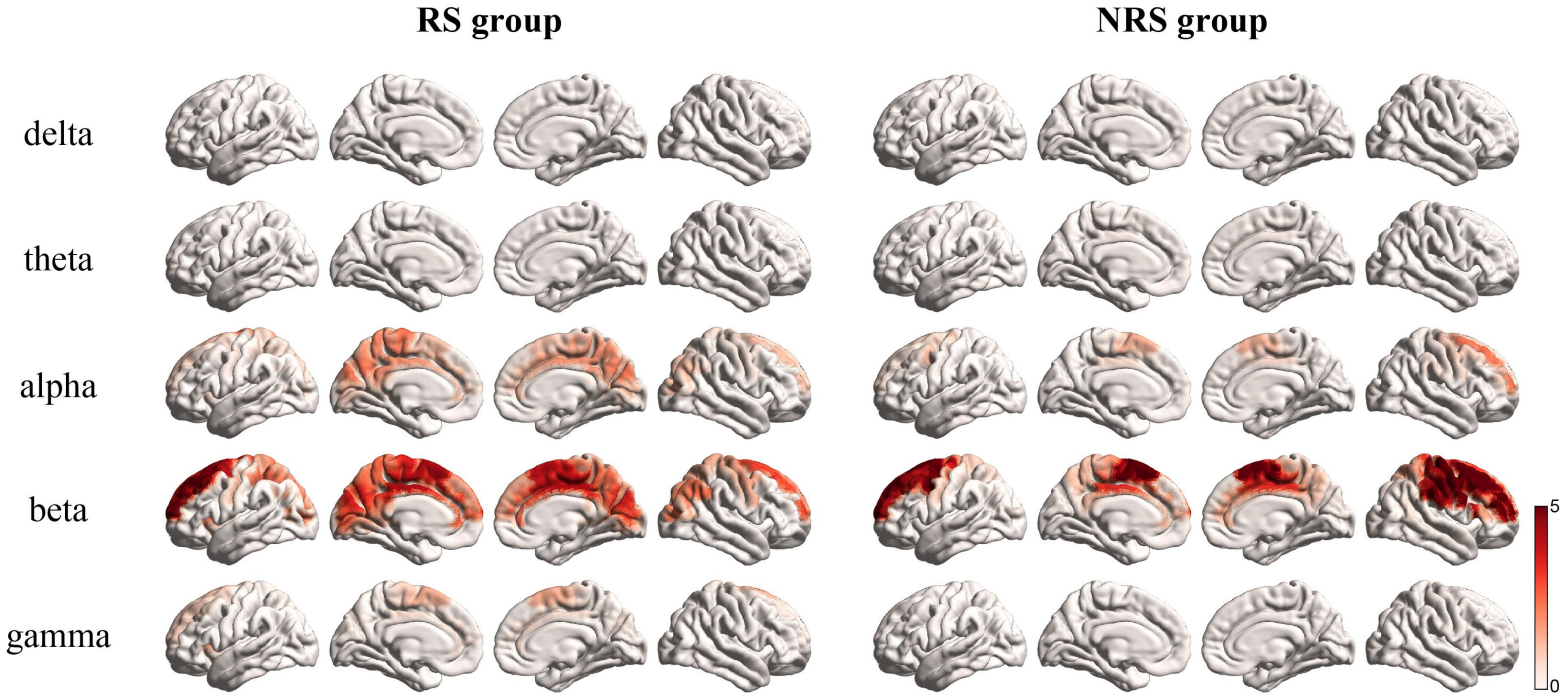
Mean brain topography scores in delta, theta, alpha, beta, and gamma rhythms for the RS and NRS groups are shown in the figure.

Earlier studies on brain rhythmic activity have consistently demonstrated that different frequency bands respond to various cognitive processes (Dai et al., 2017; Schoffelen et al., 2017). A comparison was made between the activation of the brain network of the RS and NRS groups in five different frequency bands; Delta 1–3 Hz, Theta 4–7 Hz, Alpha 8–12 Hz, Beta 13–30 Hz, and Gamma 31–60 Hz bands (Schoffelen et al., 2017). As shown in Figure 4, analysis of the frequency band rhythm distribution revealed that the brain network in the RS group was mainly active in the Alpha and Beta bands, with a small distribution in the Gamma band. The Delta and Theta bands, conversely, showed negligible activity. The NRS group brain network, likewise, exhibited maximum activity in the Beta band, with minor involvement in the Alpha band. Consistent with the RS group, the activity in the Delta and Theta bands was insignificant. However, the NRS group did not demonstrate any significant activity in the Gamma band. It is apparent from the analysis that semantically inconsistent NRS sentences, in comparison to the semantically coherent RS sentences, show higher concentration and activity in the frequency band distribution.

**Figure 5:**
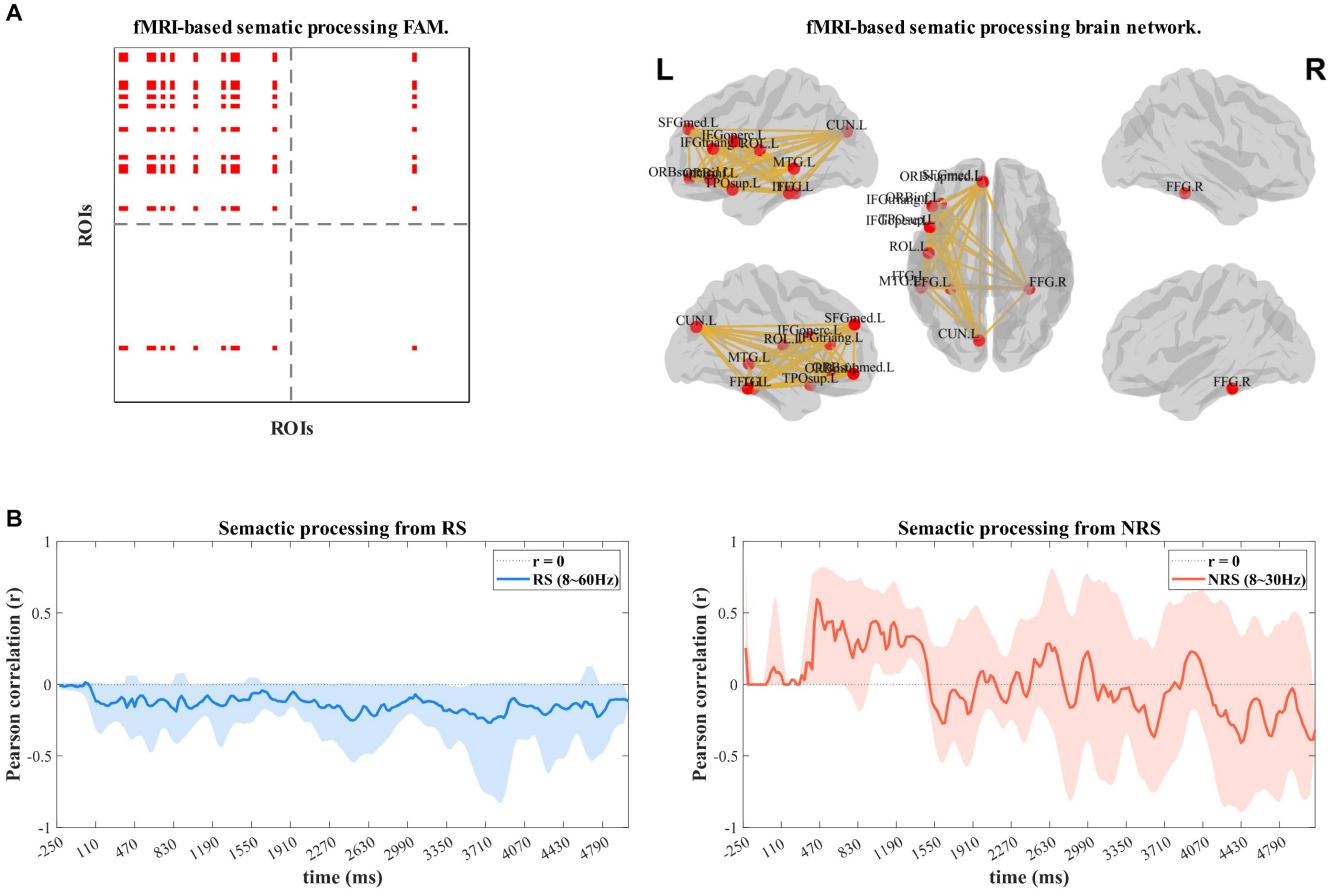
**(A)** Shows the activation FAM (functional adjacency matrices) of the semantic processing network built on the basis of the fMRI *a priori* database in the left view; the right view shows the structure of the visualization of the network. **(B,C)** Shows the results of the dynamic time-frequency-spatial correlation analysis of RS and NRS with the semantic brain network template.

The dynamic time-frequency correlation analysis method utilizing EEG brain network and fMRI functional template network (Figure 5A) involves calculating Pearson correlation coefficients of the spatially filtered semantic functional template network. The frequency range for the RS group was 8–60 Hz, including the Alpha, Beta, and Gamma bands, while the NRS group was limited to the Alpha and Beta bands. Figures 5B,C display the outcomes. The analysis of the RS group indicated a negative correlation (*r* = −0.211) between the EEG dynamic brain network and semantic functional network during continuous speech production. This negative correlation suggests that the semantic brain networks are less active and almost inactive during this process. In contrast, the analysis of the NRS group revealed a significant positive correlation (*r* = 0.349) between the EEG dynamic brain network and semantic functional network from 0 to 1,300 ms, followed by a quick decrease at about 1,400 ms (*r* = 0.334 to *r* = −0.272). The correlation gradually increased up to 2,600 ms (*r* = 0.283), then gradually decreased from 2,600 ms (*r* = 0.207) to 3,500 ms (*r* = −0.368). After that, the correlation increased again to 3,900 ms (*r* = 0.228), followed by a gradual decrease to −0.178. The findings reveal that the semantic network is prominently implicated in speech planning during the entire NRS task process. The network is recurrently activated and inhibited, demonstrating an overall inhibitory pattern.

## 4. Discussion

This study aimed to clarify the speech planning mechanisms during continuous speech production by measuring behavior (eye movement and speech) and neurophysiological (EEG) data while conducting continuous sentence reading. By controlling the semantic consistency of sentences, we discussed the semantic processing mechanism in speech planning. For each sentence, the statistical analysis was conducted on a per-word basis using the start timescales obtained from the analysis of eye movement and speech data, specifically VP, OP, SP, and EVS. Subsequently, we compared the brain activity between semantic consistency (RS) and semantic inconsistency (NRS), and analyzed its distribution across various frequency ranges. Lastly, we analyzed the dynamic process of semantic processing between two sentences based on the frequency range of distribution using dynamic spatial correlation analysis at a temporal frequency. As expected, the semantic network plays a critical role in the production of continuous speech. Furthermore, our findings suggest two distinct modes of speech production.

In a word-by-word analysis during speech production revealed that “look ahead” was prevalent in both the RS and NRS groups. Our results showed that the OP was always present and positive in both the RS and NRS. The implication of this result was that speakers access the latter lexical representations and phonological information before the speech onset of the current words. Hereby, by delaying the production of the first word in utterance until they had some sense of the availability of the phonological form of the upcoming word. This again demonstrates the expected effect of the speech execution process (Ortmanns et al., 1996; Laubrock and Kliegl, 2015). That is, the production system to achieve fluently, speakers must scan ahead to determine which sounds to produce next. This suggests that the speakers planning ahead for upcoming words by gazing ahead to the next word. This benefit is obvious, as it can effectively avoiding mispronunciation and avoid becoming tongue-tied allows for fluent speech production.

One of the significant differences between the RS and NRS groups is that the OP and EVS manifests two different tendencies. With the exception of initial words, all subsequent words showed significant differences. In the RS group, looking ahead to plan seems to become gradually less significant. From the second word onward, planning for subsequent words no longer seems to require an ocular gaze. At the end of words, OP is reduced by 76%. Therefore, it can be inferred that immediately after gazing at current words, the articulatory system is ready to execute the pronunciation of those words. This phenomenon may be attributed to the high degree of semantic coherence. The speech planning process is accelerated to the extent that the brain, comparable to ocular movements, is able to keep pace with the eyes. Eye-voice span (EVS) difference at the beginning was close to two words and reduced to one at the end. In the NRS group, the initial OP decreased slightly at the second word and increased gradually until the end of the sentence. Conversely, in the final words of the NRS group, OP and EVS increased by approximately 44 and 29% compared to initial words. The EVS covers the complete two-word span of the current and subsequent words.

Moreover, the absence of a significant difference in FFD suggests that there was no dissimilarity in the initial stages of lexical retrieval between the two groups of sentences. Previous research has demonstrated that the duration of first fixation can be affected by word length and word frequency. In our sentences, all the words have the same length and are high in frequency. Thus, the dissimilarities between GD, MFD, and EVS might be attributed to fixation points other than FFD, such as second and subsequent fixations as well as refixations. These fixation points might be engaged in higher-level lexical processing mechanisms, including semantic understanding and prediction. These higher levels of information processing are likely accountable for the discrepancies in eye movement patterns between RS and NRS.

Previous studies have established that semantics is a key factor influencing speech planning in sentence reading (Clifton et al., 2007; Pickering and Gambi, 2018). This implies that cohesive contextual semantic information hastens the speaker’s speech planning process (Huang et al., 2019), enabling them to anticipate the next speech position through semantic prediction. On the other hand, semantic inconsistency causes the speaker to invest more resources in scrutinizing and compensating for speech planning issues, ultimately impacting speech fluency. Comparing the outcomes of RS and NRS experiments, it is clear that smooth processing of semantic information is critical for continuous speech planning. Pronunciation is delayed when semantic information of subsequent words differs significantly from anticipated semantic principles. The delay guarantees accurate pronunciation devoid of tongue-tied speech. Conversely, if the semantic information of the following words is consistent with the predicted semantic information, the speaker’s pronunciation is fast and effortless, with negligible errors.

The neural data analysis results suggest a distinct dorsal flow of information during continuous speech production when there is semantic coherence. This information flow originates from the occipital lobe, passes through the parietal lobe, the central anterior–posterior gyrus, and finally reaches the frontal lobe. Although there was no significant ventral flow of information, this result partially supports the dual stream model of speech production, as this model proposes that dorsal information flow processes non-semantic pathways. This pathway is primarily responsible for the representation of phonological information processing to articulatory execution (Hickok and Poeppel, 2007). Our interpretation of this result is that the ventral pathway of semantic processing becomes less important when semantics are predictable. As a result, the speaker can continuously plan and output speech segments by prediction without the need to achieve lexical fluency. The dynamic correlation analysis results of the brain’s semantic processing network confirms this point, as the semantic processing network is negatively correlated and continuously suppressed when there is semantic coherence. However, when semantics become incoherent, the dorsal information flow becomes less significant, and the frontal lobe displays significant activation. It is well-documented that the frontal lobes are strongly associated with attention (Corbetta and Shulman, 2002; Doricchi et al., 2010), working memory (du Boisgueheneuc et al., 2006), dyslexia (Vasic et al., 2008), and other linguistic cognitive functions. In particular, the middle frontal gyrus is highly activated during Chinese reading tasks (Yan et al., 2021). Semantic incoherence increases the cognitive load of the language task, resulting in an increased need for attention allocation and more working memory consumption, which makes reading significantly more difficult. Therefore, during the NRS group, frontal lobe activation plays a significant role. Furthermore, the correlation results with the semantic processing network confirm that the activation of the semantic network is alternately positively and negatively correlated, with an overall decreasing trend. This indicates that speakers try to make sense of the sentence through semantic access to words before and after it, leading to a positive strong correlation in the early stage. However, the correlation gradually weakens because it was found that it cannot be correctly understood in the later period. The ups and downs during the reading aloud also prove that speakers have been trying to understand the sentences during continuous speech production.

The dorsal pathway in the dual-stream model is typically utilized to describe the processing of non-semantic linguistic information, whereas the ventral pathway primarily handles semantics. Through the analysis of dynamic brain networks, we have discovered that the coherence of semantics exerts differential effects on the activation of these two pathways. Specifically, coherent semantic information strengthens the activation of the dorsal pathway due to its contextual coherence, thus enhancing language prediction with minimal involvement of the ventral semantic pathway. Conversely, incoherent semantic information triggers repeated attempts to activate the ventral semantic pathway in order to disambiguate the semantics before and after and comprehend the sentence. During this process, the dorsal pathway solely furnishes basic phonological representations. Moreover, attention allocation and efforts to rectify erroneous language occur through the engagement of higher cognitive regions such as the prefrontal cortex. Overall, the predictability of semantics appears to be a potential regulator of comprehensive sentence comprehension and production. This distinction represents the most significant deviation from conventional language models, thereby warranting our focused investigation into semantic prediction in future research and analysis.

Overall, our behavioral analysis suggests that look-ahead is a prevalent phenomenon during continuous speech production. We explain the critical role of semantic continuity in the speech planning process and present the variation pattern of look-ahead magnitude. Additionally, we demonstrate that semantic understanding plays a more significant role in continuous speech production than traditional predictors, such as syllable length and word familiarity. These findings are further supported by the analysis of neural data, which reveals a dorsal information pathway consistent with the dual pathway model and a heavy involvement of the frontal lobes during semantic discontinuity. Our study contributes to filling the gap of speech planning mechanisms during continuous speech production and provides a feasible research method for future studies in this field.

## 5. Conclusion

Our study seeks to explore the time variation and brain semantic network in continuous speech planning using behavioral and neuroscientific analyses. Based on our behavioral findings, we found that semantic predictability is a fundamental factor influencing the reduction in latency during continuous speech planning. In the presence of predictable sentence semantics, the degree of lookahead tends to decrease as the understanding of the sentence unfolds. Conversely, when semantic incoherence occurs, the eye-voice span continues to cover two words. In addition, we observed that the correlation between the brain’s semantic processing network and semantic comprehension is strong during difficult comprehension tasks compared to easier ones. In conclusion, co-registering EEG, speech, and eye movements in continuous speech can be an effective way to gain insight into the brain’s speech planning process from fixation onset to speech onset. It can also provide a dynamic representation of the discourse planning process with high spatial and temporal resolution.

## Data availability statement

The raw data supporting the conclusions of this article will be made available by the authors, without undue reservation.

## Ethics statement

The studies involving humans were approved by Tianjin University Research Ethics Committee Japan Advanced Institute of Science and Technology Research Ethics Committee. The studies were conducted in accordance with the local legislation and institutional requirements. The participants provided their written informed consent to participate in this study. The animal study was approved by Tianjin University Research Ethics Committee Japan Advanced Institute of Science and Technology Research Ethics Committee. The study was conducted in accordance with the local legislation and institutional requirements.

## Author contributions

JH conducted the experiments, analyzed the data, interpreted the findings, and prepared the manuscript. GZ conceptualized the research idea, provided the experimental platform, and revised the manuscript. JD contributed to the research idea, provided an experimental platform, and revised the manuscript. YC critically reviewed and revised the manuscript. SM contributed to the research idea and revised the manuscript. All authors contributed to the article and approved the submitted version.

## Funding

This research was supported by the National Natural Science Foundation of China (Nos. 61876126 and 62276185) and partially funded by JSPS KAKENHI Grants (Nos. 20K11883 and 22J10733).

## Conflict of interest

JH was employed by company NeuralEcho Technology Co., Ltd.

The remaining authors declare that the research was conducted in the absence of any commercial or financial relationships that could be construed as a potential conflict of interest.

## Publisher’s note

All claims expressed in this article are solely those of the authors and do not necessarily represent those of their affiliated organizations, or those of the publisher, the editors and the reviewers. Any product that may be evaluated in this article, or claim that may be made by its manufacturer, is not guaranteed or endorsed by the publisher.

## Figures and Tables

**Table:** 

EEG	Electroencephalography
EVS	Eye-voice span
RS	Regular sentences
NRS	Nonregular sentences
EOG	Electrooculogram
FFD	First fixation duration
MFD	Mean fixation duration
GD	Gaze duration
GOn	Gaze onset
GOf	Gaze offset
SOn	Speech pronunciation onset
SOf	Speech pronunciation offset
VP	Viewing period
OP	Overlapping period
SP	Speech period
FAM	Functional adjacency matrices
ICA	Independent component analysis
AMICA	Adaptive mixture independent component analysis algorithm
MRI	Magnetic resonance imaging
fMRI	Functional MRI
SIFT	Source information flow toolbox
